# Association Between Professional Self-Efficacy and Self-Reported Compassionate Communication Among Nurses: A Cross-Sectional Study with Exploratory Analyses of Generative Altruism Scale Scores

**DOI:** 10.3390/healthcare14213608

**Published:** 2026-10-25

**Authors:** Havva Akpınar

**Affiliations:** Department of Psychiatric Nursing, Faculty of Health Sciences, Mugla Sıtkı Kocman University, Mugla 48000, Türkiye; havvaakpinar@mu.edu.tr

**Keywords:** compassionate communication, generative altruism, nurses, professional self-efficacy, cross-sectional study

## Abstract

**Highlights:**

**What are the main findings?**

- Scores on the Nursing Profession Self-Efficacy Scale (NPSE) and Compassionate Communication Scale (CCS) were positively associated after covariate adjustment; sensitivity analyses yielded similar estimates.
- Neither ordinal factor model for the Generative Altruism Scale (GALS) fitted adequately, and total-score reliability was limited (α = 0.613), preventing substantive interpretation of its exploratory associations as evidence about generative altruism.

**What are the implications of the main findings?**

- Compassionate Communication Scale’s unusually high alpha (0.994) and identical item response patterns raise concerns about potential item redundancy and measurement validity; the association does not establish causality or differences in observed communication behavior.
- Multicenter longitudinal studies with independent communication assessments and controlled intervention research are needed before these findings can inform educational recommendations; the present findings do not justify implementing self-efficacy or generative-altruism training.

**Abstract:**

Background: Compassionate communication, professional self-efficacy, and generative altruism represent potentially related aspects of nursing practice. This study examined the adjusted association between professional self-efficacy and self-reported compassionate communication, alongside the measurement properties of the Generative Altruism Scale (GALS) and exploratory associations involving its scores. Methods: This cross-sectional study used nonprobability recruitment at one hospital in Türkiye. All analyses included 201 nurses with source-verified responses to the Compassionate Communication Scale (CCS), Nursing Profession Self-Efficacy Scale (NPSE), and Generative Altruism Scale (GALS). Hierarchical regression entered demographic and professional covariates in Block 1 and NPSE in Block 2, which constituted the primary adjusted model. GALS scores were added in an exploratory third block. Ordinal GALS factor models were estimated using weighted least squares with mean- and variance-adjusted test statistics (WLSMV). Results: CCS and NPSE scores were positively correlated (r = 0.591, *p* < 0.001). NPSE remained associated with CCS after covariate adjustment (B = 1.032, 95% CI [0.796, 1.267], *p* < 0.001). The primary adjusted model explained 52.2% of CCS-score variance; adding NPSE yielded ΔR^2^ = 0.187 (F-change (1, 190) = 74.550, *p* < 0.001). Sensitivity analyses yielded similar estimates. CCS alpha was 0.994, with identical responses within multiple item sets. Adding GALS provided no statistically significant increment (ΔR^2^ = 0.006, *p* = 0.115). Neither GALS factor model fitted adequately, and total-score reliability was limited (α = 0.613). Conclusions: The observed NPSE–CCS score association requires caution because CCS measurement validity remains uncertain. Cross-sectional self-report data do not establish causality or empirical construct distinctiveness. GALS findings do not support construct-level conclusions about generative altruism. Single-hospital recruitment and potential nonresponse bias limit generalizability. Multicenter longitudinal research with independent communication assessments is needed before educational recommendations can be made.

## 1. Introduction

Effective nurse–patient communication extends beyond exchanging clinical information and involves ethical and professional values, empathic skills, and responsiveness to patients’ individual needs [1,2]. It is central to patient-centered care and therapeutic relationships, shaping patients’ experiences of nursing care [3]. Compassion and altruism provide relevant perspectives for understanding the interpersonal and motivational aspects of these interactions [4,5].

Compassion involves recognizing another person’s suffering and responding sensitively with a willingness to alleviate it [6,7,8]. Compassionate communication expresses this orientation through verbal, nonverbal, and relational behaviors, including attentive listening, sensitive explanations, and acknowledgment of patients’ emotional needs [7,9,10]. Compassion is embedded in nursing ethics and professional practice [11], and compassion-based care has been associated with patient and nurse outcomes [12,13]. Examining compassionate communication therefore focuses attention on a specific interpersonal aspect of nursing practice rather than overall care quality or professional performance.

Nursing professional self-efficacy refers to nurses’ beliefs in their ability to fulfill professional responsibilities and manage clinical and interpersonal challenges [14,15]. Previous research has examined its relationships with holistic nursing competence and compassion-related competencies [16,17], while evidence also links professional self-efficacy with patient-communication skills [18]. These findings provide a rationale for examining whether professional self-efficacy is associated specifically with compassionate communication. Such an investigation can extend existing research to communication centered on recognizing and responding to patients’ suffering, without assuming that efficacy beliefs necessarily translate into observed behavior.

Altruism broadly refers to voluntary behavior intended to benefit others without external reward [4,19]. Generative altruism concerns the satisfaction derived from supporting other people’s development, success, and well-being [19,20,21,22]. It offers a motivational perspective that is relevant to nurses’ helping role but differs from compassion and its interpersonal expression. Its inclusion in this study permits an exploratory examination of whether scores intended to capture this orientation are associated with professional self-efficacy and compassionate communication, provided that their measurement limitations are considered.

The three concepts thus address different aspects of nursing practice: professional self-efficacy concerns competence beliefs, compassionate communication concerns interpersonal behavior, and generative altruism concerns a motivational orientation [18,20,21,23,24]. In this study, however, communication behavior was assessed by self-report rather than direct observation. Conceptual differences between these attributes do not establish that their questionnaires measure empirically distinct constructs. The analysis therefore concerns observed self-report scores, with the interpretation of Generative Altruism Scale (GALS) findings dependent on its measurement performance in this sample.

This study examined the adjusted association between nursing professional self-efficacy and self-reported compassionate communication among nurses, with secondary analyses of GALS measurement properties and exploratory associations involving its scores. In the revised analytical framework, the primary analysis examined the association between Nursing Profession Self-Efficacy Scale (NPSE) and Compassionate Communication Scale (CCS) scores after adjustment for selected demographic and professional characteristics. The reliability and factor structure of GALS were evaluated to inform interpretation of its exploratory associations. All subgroup comparisons were also considered exploratory.

The following research questions guided the investigation, with the distinction between primary and exploratory analyses clarified during revision:

1. What are the distributions of CCS, NPSE, and observed GALS scores?
2. How are CCS and NPSE scores associated, and what exploratory correlations are observed between GALS and these scores?
3. Is NPSE associated with CCS after adjustment for selected demographic and professional covariates, and does this association persist when GALS is added in a separate exploratory block?
4. What exploratory differences in these scores are observed across sociodemographic and professional subgroups?

## 2. Materials and Methods

### 2.1. Study Design

The study was designed as a descriptive and correlational cross-sectional investigation. The STROBE recommendations guided the reporting process [25].

### 2.2. Participants and Procedure

The source population comprised 335 nurses working in authorized clinical units other than the operating room at a training and research hospital in Türkiye during the data collection period from 1 April to 31 May 2023 (N = 335). Operating-room nurses were outside the accessible study population because institutional authorization for data collection in that unit was not granted due to infection-control restrictions. Nurses employed in the authorized clinical units who voluntarily provided informed consent were eligible to participate.

The initial sample-size requirement was estimated using a finite-population formula for estimating a proportion. Assuming an expected proportion of 50%, a 95% confidence level, and a 5% margin of error, the minimum required sample was calculated as 179 nurses [26]. This number was increased by 10% to allow for potential nonparticipation or unusable questionnaires, resulting in a recruitment target of 197 nurses [26]. This calculation established the recruitment target rather than prospective power for the correlation or regression analyses. All 335 eligible nurses were invited to participate; recruitment was nonprobability-based, with participation determined by voluntary consent and questionnaire completion. A total of 201 nurses participated, corresponding to a participation rate of 60.0%. The remaining 134 eligible nurses did not participate or did not return a questionnaire. No participant withdrew consent after enrollment, and no questionnaire was excluded in its entirety as incomplete or unusable.

The electronic responses to all 23 CCS items were compared item by item with the corresponding responses on the original paper questionnaires for each of the 201 participants. No CCS item responses were changed. Identical responses within four CCS item sets (Items 1–3, 8–10, 12–13, and 19–20) were present in both the paper questionnaires and the electronic records for every participant. This verification confirmed agreement between the source questionnaires and the electronic data; it did not establish measurement validity. Following source verification, complete item-level data for the CCS, GALS, and NPSE were available for all 201 participants and were used for the descriptive, psychometric, correlational, and regression analyses. No missing values were imputed.

After the achieved sample size was known, a sensitivity analysis was conducted to estimate the incremental effect size detectable in the revised primary regression model. The calculation used the noncentral F distribution implemented in SciPy version 1.17.0 [27], with 201 participants, one tested predictor (NPSE), 10 predictors in the full model (nine covariate indicators and NPSE), and α = 0.05. The minimum detectable incremental effect size was f^2^ = 0.039 at 80% power and f^2^ = 0.053 at 90% power. This analysis describes sensitivity conditional on the available sample and specified regression model; it does not establish that the study was prospectively powered for this model. It also does not justify the exploratory subgroup comparisons, particularly those involving small or highly unbalanced groups.

### 2.3. Data Collection Instruments

The data collection instruments comprised a Descriptive Information Form, the Compassionate Communication Scale (CCS), the Generative Altruism Scale (GALS), and the Nursing Profession Self-Efficacy Scale (NPSE).

#### 2.3.1. Descriptive Information Form

The Descriptive Information Form was developed by the researcher based on previous studies addressing the study constructs [6,7,8,9,10]. It comprised 13 questions covering age, gender, marital status, educational level, professional experience, clinical unit, perceived professional competence in nursing, previous communication-related training, familiarity with the term “compassionate communication,” willingness to receive training in compassionate communication, previous exposure to the term “generative altruism,” previous generative altruism-related training, and willingness to receive training in generative altruism.

#### 2.3.2. Compassionate Communication Scale

Ramos Salazar [10] developed the Compassionate Communication Scale (CCS), and İbrahimoğlu et al. [9] subsequently evaluated the psychometric properties of its Turkish version. The instrument comprises 23 items rated from 1 (never) to 5 (always). Item scores are summed to obtain a total score ranging from 23 to 115, with higher scores indicating more frequent self-reported compassionate communication. The Turkish adaptation reported a Cronbach’s alpha of 0.94 [9]. In the present sample, Cronbach’s alpha calculated from the 23 items after verification against the original paper questionnaires was 0.994 (n = 201). The unusually high coefficient and identical responses within several item sets raise concerns about potential item redundancy. This internal-consistency estimate does not establish measurement validity.

#### 2.3.3. Generative Altruism Scale

Büssing et al. developed the Generative Altruism Scale (GALS) to assess generative altruism [20]. Anlı subsequently adapted the scale into Turkish and reported a nine-item, two-factor structure with satisfactory psychometric properties [22]. Items are rated from 0 (never) to 3 (very often), yielding a total score from 0 to 27. Higher scores are intended to reflect greater generative altruism. Cronbach’s alpha was 0.82 in the Turkish adaptation study [22].

In the present study, reliability was recalculated from the verified item responses of all 201 participants using the original 0–3 coding, without reverse coding or imputation. Cronbach’s alpha was 0.613 for the total score, 0.434 for the item group comprising Items 1–3 and 9, and 0.498 for the item group comprising Items 4–8. The published correlated two-factor structure and a competing one-factor model were evaluated using the procedures described in the Section 2.5.

Given the limited internal consistency and inadequate model fit in this sample, GALS totals were retained only as exploratory observed scores. The proposed dimensions were not used as separate outcomes. Associations involving GALS scores were not interpreted as evidence of relationships involving an adequately measured generative altruism construct, and nonsignificant findings were not taken as evidence of an absence of such relationships.

#### 2.3.4. Nursing Profession Self-Efficacy Scale

Caruso et al. developed the Nursing Profession Self-Efficacy Scale (NPSE) to measure nurses’ confidence in managing professional responsibilities and challenges [15]. Kacaroğlu Vicdan and Taştekin subsequently adapted the instrument for use in Türkiye [14]. The Turkish version contains 16 positively worded items; each is scored from 1 (strongly disagree) to 5 (strongly agree). The summed score ranges from 16 to 80, and higher values reflect stronger nursing professional self-efficacy. Cronbach’s α was 0.87 in the Turkish adaptation [14] and 0.91 in the current sample.

### 2.4. Data Collection

The researcher collected the data in person at the hospital using paper-based questionnaires; no online data-collection method was used. Nurses who met the eligibility criteria were approached at suitable times during their work schedules and were informed about the study before deciding whether to participate. After providing written informed consent, participants completed the questionnaires in approximately 10 min. Data collection was scheduled to avoid interference with clinical duties and patient care and was conducted in accordance with institutional procedures, infection-control requirements, ethical standards, and patient rights.

No definition or detailed explanation of generative altruism was provided before participants answered the corresponding descriptive question, as this could have influenced their reporting of previous exposure to the concept. The question asked whether participants had previously heard of the concept of generative altruism and did not assess their knowledge or conceptual understanding. All participants received the same written question and standardized response options. Participants subsequently completed the Turkish version of the GALS without additional explanation or interpretation by the researcher.

### 2.5. Statistical Analysis

Initial data management and statistical analyses were performed using IBM SPSS Statistics version 25. Analyses were subsequently recalculated from the verified item responses using Python version 3.12.14 with the SciPy and statsmodels libraries. Ordinal GALS factor models were estimated using lavaan version 0.7-2 in R version 4.5.3. Descriptive statistics included frequencies, percentages, means, and standard deviations or medians and interquartile ranges, as appropriate. Pearson correlations were calculated among the observed CCS, NPSE, and GALS total scores, with 95% confidence intervals obtained using Fisher’s z transformation. All analyses included the same 201 participants; no missing values were imputed.

The primary analysis examined the adjusted association between NPSE and CCS using hierarchical multiple linear regression, with the CCS total score as the dependent variable. Block 1 included age group, gender, professional experience group, clinical unit, perceived professional competence, and previous communication-related training. These covariates represented demographic characteristics, professional exposure, clinical context, perceived competence, and prior educational exposure. They were selected on theoretical grounds rather than solely according to statistical significance in exploratory subgroup comparisons. NPSE was entered in Block 2, which constituted the primary adjusted model. GALS total scores were added in Block 3 as a separate exploratory extension. This block structure was adopted during revision to distinguish the primary NPSE–CCS analysis from exploratory analyses involving GALS scores.

For subgroup comparisons and the main regression models, age was categorized into approximately 10-year intervals across the observed range: 22–31, 32–41, and ≥42 years. Professional experience was recorded in three categories (<1 year, 1–5 years, and ≥6 years), corresponding to the initial transition, early-career, and more established experience periods. Both variables were entered as categorical indicators rather than numeric group codes. The six covariates contributed nine indicator variables to Block 1. Reference categories were age 22–31 years, female gender, ≥6 years of experience, medical units, reporting professional competence, and having received previous communication-related training. Marital status was omitted because its additional theoretical contribution after accounting for age and experience was considered less direct. Educational level was omitted because of its highly unbalanced distribution, particularly the small postgraduate group.

Regression results were summarized using unstandardized coefficients, standard errors, standardized coefficients, 95% confidence intervals, p values, R^2^, adjusted R^2^, and changes in R^2^ with corresponding F-change tests. The relationship between perceived professional competence and NPSE was examined using a point-biserial correlation, with competence coded as 1 = yes and 0 = no. Sensitivity analyses repeated the primary adjusted model (Block 2) after excluding perceived professional competence and, separately, after replacing age categories with age in years as a continuous variable while retaining all other covariates. The primary Block 2 model was also evaluated using HC3 heteroscedasticity-robust standard errors.

Diagnostics for the primary Block 2 model included the Ramsey RESET test with a squared fitted-value term for functional-form misspecification, the Shapiro–Wilk test for residual normality, and the studentized Breusch–Pagan (Koenker) test for heteroscedasticity. Multicollinearity was examined using variance inflation factors and tolerance values, and potentially influential observations were assessed using Cook’s distance. Normality-test results were interpreted cautiously because test performance varies with sample size and distributional characteristics [28]. These diagnostics were considered together rather than treated as definitive evidence that all model assumptions were satisfied.

GALS measurement analyses compared the published correlated two-factor structure, comprising Items 1–3 and 9 and Items 4–8, with a competing one-factor model. All nine items were specified as ordered categorical variables, and both models were estimated using weighted least squares with mean- and variance-adjusted test statistics (WLSMV), reflecting the four-category response format. Delta parameterization was used, with latent factor variances fixed to one. Original 0–3 coding was retained; no items were removed, response categories combined, or residual covariances added. Thresholds were estimated for the categories observed in the sample.

Model evaluation considered the adjusted chi-square statistic, comparative fit index (CFI), Tucker–Lewis index (TLI), root mean square error of approximation (RMSEA) with 95% confidence intervals, and standardized root mean square residual (SRMR). Scaled and robust fit indices were reported separately. Model fit was compared descriptively, without a formal chi-square difference test. Cronbach’s alpha was recalculated for the GALS total score and both published item groups using the original numerically coded responses rather than the polychoric correlation matrix. No alternative scoring scheme was developed. GALS associations were treated as exploratory observed-score findings because of the scale’s inadequate measurement performance in this sample.

All subgroup comparisons across CCS, NPSE, and GALS were designated as exploratory. The test selected for each comparison in the original analysis was retained when recalculating results from the verified data. These tests comprised Student’s or Welch’s independent-samples t-tests, conventional or Welch’s one-way analysis of variance, and Kruskal–Wallis or Mann–Whitney U tests. Comparisons by educational level, professional experience, previous awareness of generative altruism, and willingness to receive generative-altruism training used nonparametric methods because of particularly small or unbalanced groups. Nonparametric comparisons were summarized using medians and first and third quartiles. Mann–Whitney statistics were reported as U_1_ for the first listed group, with two-sided asymptotic p values incorporating tie and continuity corrections. Kruskal–Wallis statistics included tie correction.

For nominally significant three-group omnibus tests, pairwise comparisons used Tukey’s honestly significant difference test after conventional analysis of variance, Games–Howell tests after Welch’s analysis of variance, or Dunn’s tests with Bonferroni adjustment after Kruskal–Wallis tests. These adjustments were applied within each comparison family and did not control multiplicity across the full set of exploratory analyses.

Effect sizes comprised pooled-standard-deviation Cohen’s d for parametric two-group comparisons, conventional ANOVA omega-squared (ω^2^) for parametric three-group comparisons, rank-biserial correlations for Mann–Whitney U tests, and Kruskal–Wallis epsilon-squared (ε^2^ = H/[N − 1]). Conventional ω^2^ described between-group variation and was not a Welch-derived effect size. Pointwise 95% confidence intervals for all effect-size estimates were calculated using a group-stratified percentile bootstrap with 10,000 resamples, preserving the original group sizes. The definitions, direction of effects, and bootstrap procedure are detailed in Supplementary Table S1. These intervals were not adjusted for multiplicity, did not directly invert the reported hypothesis tests, and were interpreted cautiously for small groups and discrete distributions.

All 36 subgroup comparisons, together with effect sizes and confidence intervals, were consolidated in Supplementary Table S1. No global multiplicity correction was applied. Interpretation emphasized effect magnitude, uncertainty, and group size rather than nominal statistical significance, and findings from very small subgroups were not interpreted substantively. All tests were two-tailed, with *p* < 0.05 used as the nominal significance threshold [29,30].

### 2.6. Ethical Considerations

Ethical clearance was granted by the Health Sciences Ethics Committee of a public university before participant recruitment. Administrative approval from the hospital and permission to use the study instruments were also secured. Before enrolment, nurses received written information concerning the study’s purpose, procedures, voluntary nature, confidentiality safeguards, and their right to withdraw without any adverse consequences. Written informed consent was obtained from all participants. No names or other direct personal identifiers were recorded, and the research data were handled and stored confidentially. The study procedures complied with the ethical principles of the Declaration of Helsinki applicable during the study period.

## 3. Results

The descriptive characteristics of the participating nurses are presented in Table 1. Their mean age was 36.90 ± 8.93 years. Of the participants, 88.6% were women, 68.2% were married, 86.6% held a bachelor’s degree, 75.1% had six or more years of professional experience, and 41.3% worked in internal medical units. Furthermore, 86.6% perceived themselves as professionally competent in nursing. Most participants had not received previous communication-related education or training (67.7%), and 67.2% reported that they were not familiar with the term compassionate communication. In addition, 96.0% had not previously heard of the concept of generative altruism, and none had received previous education or training related to generative altruism. Regarding self-reported educational interest, 80.6% expressed willingness to receive training in compassionate communication and 96.0% expressed willingness to receive training in generative altruism.

The mean total scale scores are presented in Table 2. Mean scores were 91.14 ± 14.63 for the CCS, 8.35 ± 2.57 for the GALS, and 63.86 ± 6.75 for the NPSE. Observed GALS scores ranged from 3 to 17 within the theoretical range of 0–27. No participant obtained either theoretical endpoint; however, this observation alone does not establish the absence of floor or ceiling effects. The observed score range was narrower than the theoretical range, with a mean below the theoretical midpoint.

The GALS measurement evaluation included all 201 participants (Table 3). In the ordinal WLSMV analysis, the correlated two-factor model showed inadequate fit: adjusted χ^2^(26) = 93.245, *p* < 0.001, scaled CFI = 0.866, scaled TLI = 0.815, scaled RMSEA = 0.114 (95% CI [0.084, 0.144]), and SRMR = 0.120. The one-factor model showed poorer descriptive fit: adjusted χ^2^(27) = 119.535, *p* < 0.001, scaled CFI = 0.816, scaled TLI = 0.754, scaled RMSEA = 0.131 (95% CI 0 [0.103, 0.160]), and SRMR = 0.133. Additional robust fit indices also indicated inadequate fit for both models (Table 3). Both models converged without warnings or negative residual variances. In the two-factor model, the standardized loading for Item 9 was 0.163 (95% CI [−0.010, 0.336], *p* = 0.065), and the latent-factor correlation was 0.648. Cronbach’s alpha was 0.613 for the total score, 0.434 for Items 1–3 and 9, and 0.498 for Items 4–8. Several response categories were sparse or unobserved. Overall, neither model provided adequate factorial support for interpreting GALS scores as a measure of generative altruism in this sample.

Exploratory comparisons of CCS, NPSE, and observed GALS scores across sociodemographic and professional subgroups are presented in Supplementary Table S1, together with group sizes, effect-size estimates, and 95% confidence intervals. These comparisons were not adjusted for multiplicity across the full set of tests. Findings involving small or highly unbalanced groups were not interpreted substantively, and GALS comparisons were restricted to observed scores because of the scale’s measurement limitations.

The CCS and NPSE total scores were positively correlated (r = 0.591, 95% CI [0.493, 0.674], *p* < 0.001). Exploratory correlations of GALS scores with CCS (r = 0.028, 95% CI [−0.111, 0.166], *p* = 0.695) and NPSE (r = 0.123, 95% CI [−0.016, 0.257], *p* = 0.083) were not statistically significant (Table 4). Given the measurement limitations of GALS, these findings should not be interpreted as evidence of an absence of relationships involving the underlying generative altruism construct.

The hierarchical regression analysis included all 201 participants (Table 5). Demographic and professional covariates entered in Model 1 accounted for 33.5% of the variance in CCS scores (R^2^ = 0.335; adjusted R^2^ = 0.304). Adding NPSE in Model 2 increased the explained variance to 52.2% (R^2^ = 0.522; adjusted R^2^ = 0.497), with an incremental contribution of 18.7 percentage points (ΔR^2^ = 0.187; F-change (1, 190) = 74.550, *p* < 0.001). NPSE was positively associated with CCS after covariate adjustment (B = 1.032, SE = 0.119, β = 0.476, 95% CI [0.796, 1.267], *p* < 0.001).

Adding GALS in the exploratory third block yielded R^2^ = 0.529 and adjusted R^2^ = 0.501. The additional explained variance was not statistically significant (ΔR^2^ = 0.006; F-change (1, 189) = 2.507, *p* = 0.115). The GALS coefficient was B = 0.519 (95% CI [−0.128, 1.166], *p* = 0.115), while the adjusted NPSE–CCS association remained positive (B = 0.988, β = 0.456, 95% CI [0.748, 1.229], *p* < 0.001). GALS findings concern observed scores and should be interpreted in light of the scale’s inadequate measurement properties.

Sensitivity analyses. When age was entered as a continuous variable instead of age categories, the adjusted association between NPSE and CCS remained similar (B = 1.031, 95% CI [0.797, 1.266], *p* < 0.001). This model explained 52.1% of the variance in CCS scores (R^2^ = 0.521; adjusted R^2^ = 0.498). Adding NPSE after the demographic and professional covariates yielded ΔR^2^ = 0.188 (F-change (1, 191) = 75.072, *p* < 0.001), indicating similar estimates under the two specifications of age.

Perceived professional competence, coded as 1 = yes and 0 = no, was positively correlated with NPSE scores (point-biserial r = 0.221, *p* = 0.002). Excluding perceived professional competence from the primary adjusted model yielded a similar NPSE coefficient (B = 1.031, 95% CI [0.788, 1.273], *p* < 0.001). Using HC3 heteroscedasticity-robust standard errors for the primary adjusted model also supported the positive NPSE–CCS association (B = 1.032, 95% CI [0.789, 1.274], *p* < 0.001). These findings indicate that the estimated association was stable when perceived professional competence was excluded and when robust standard errors were used.

For the primary adjusted model (Model 2), the Shapiro–Wilk test of residuals yielded W = 0.991 (*p* = 0.284), the studentized Breusch–Pagan test yielded χ^2^(10) = 9.268 (*p* = 0.507), and the Ramsey RESET test with a squared fitted-value term yielded F(1, 189) = 0.473 (*p* = 0.492). Variance inflation factors ranged from 1.210 to 2.891, and tolerance values ranged from 0.346 to 0.827. The maximum Cook’s distance was 0.218. These diagnostics were considered alongside the HC3 sensitivity analysis.

## 4. Discussion

To examine how nurses’ professional capability beliefs relate to the interpersonal aspects of care, this study assessed the adjusted NPSE–CCS association, alongside GALS measurement properties and exploratory score associations. In this single-hospital sample, NPSE and CCS scores were positively associated after covariate adjustment. The primary model accounted for 52.2% of CCS-score variance, with an increase of 18.7 percentage points after NPSE was added. These statistics concern observed self-report scores and do not quantify an effect on communication behavior. Sensitivity analyses yielded similar estimates under the specifications examined, but this consistency does not resolve CCS measurement concerns or establish causality. Adding GALS yielded no statistically significant increment (ΔR^2^ = 0.006, *p* = 0.115); its measurement limitations preclude interpreting this as evidence that generative altruism is unrelated to compassionate communication.

The mean CCS score was 91.14 ± 14.63 (approximately 3.96 per item out of 5), placing responses toward the scale’s upper portion without establishing a validated “high” category. Compassionate communication is a specific interpersonal expression of compassion [5,9,23,31]. Güdük et al. [32] assessed general compassion among nurses and nursing students using the Compassion Scale, limiting direct comparison.

Compassionate care includes attentive listening, clear explanations, reassurance, and sensitivity to individual needs [7]. Therapeutic communication skills [3] share relational features with compassionate communication but are not interchangeable. Education and practice may support compassion-related communication [23], although barriers include insufficient formal education [8], heavy workloads, limited managerial support, and an unsupportive team culture [33]. This literature supports educational research without establishing effectiveness in the present sample.

GALS scores averaged 8.35 ± 2.57 (n = 201; observed range, 3–17; theoretical range, 0–27) and describe instrument responses rather than levels of generative altruism. Absence of scores at the theoretical endpoints does not rule out floor or ceiling effects. The Turkish version was initially evaluated in adolescents [22]; transfer to adult nurses requires validation. Neither the published two-factor nor the competing one-factor ordinal WLSMV model fitted adequately. Reliability was limited for the total score (α = 0.613) and published item groups (0.434 and 0.498). These findings indicate a sample-specific measurement problem. Kıvanç et al. [34] used the Altruism Scale and Compassion Scale to examine altruism–compassion associations in nurses; differences in measurement limit direct comparison.

The mean NPSE score was 63.86 ± 6.75 (approximately 3.99 per item out of 5), which describes response location rather than a validated “high” category. Using the same instrument, Durmus and Akpinar [16] reported 64.79 ± 11.24 among nurses in Türkiye.

Studies have examined professional self-efficacy in relation to care quality, patient safety, and nursing competence [16]. In a cross-sectional comparison, Weil-Lotan et al. [35] found higher self-efficacy among nurse educators than clinical nurses, with professional experience and postgraduate specialization also associated with self-efficacy. Bakhtiari-Dovvombaygi et al. [36] reported a positive association between the NPSE “support situation” subscale and self-reported caring behaviors in critical care nurses.

These findings and work on efficacy beliefs and caring competencies [16,17] provide context for the present association between professional capability beliefs and reported interpersonal behavior. The constructs differ conceptually, but their empirical distinctiveness was not established here. Al Wali et al. [37] examined creative self-efficacy, innovative work behavior, and job performance among physicians; these different constructs and populations provide only indirect context.

Exploratory correlations of GALS scores with NPSE and CCS were not statistically significant. However, the limited reliability and inadequate factorial support for GALS in this sample preclude interpreting these findings as evidence that generative altruism is unrelated to professional self-efficacy or compassionate communication. Studies using other altruism measures or populations [20,38] cannot resolve the current measurement limitations. Qualitative work by Alavi et al. [39] identified altruism as a values-related dimension of caring self-efficacy among Iranian pediatric nurses and nursing educators. In a concept clarification study, Slettmyr et al. [40] characterized professional altruism as a moral orientation in nursing care and part of professional identity. A nursing-student study using another altruism measure reported a positive association with compassionate communication [41]. These findings provide conceptual and empirical context, but independent GALS validation in adult nurses is required before interpreting its scores as generative altruism.

Most participants were unfamiliar with the term “compassionate communication” (67.2%) and the concept of generative altruism (96.0%), while 80.6% and 96.0%, respectively, expressed interest in training. Terminology familiarity does not measure knowledge, communication skills, or compassionate practice, and willingness to train establishes neither educational need nor effectiveness. Multicenter longitudinal studies using patient or colleague assessments and independent observations could examine whether changes in professional self-efficacy precede changes in compassionate communication. Subsequent controlled intervention research would be needed to support educational recommendations; the present findings do not justify implementing self-efficacy or generative-altruism training.

Methodological strengths include examining the NPSE–CCS association after adjustment for selected demographic and professional covariates. Consistent estimates across alternative model specifications and analyses using HC3 robust standard errors supported statistical stability within the examined specifications, while measurement validity remained unresolved. Verification against the original paper questionnaires provided a direct check of electronic item responses, accompanied by transparent reporting of repeated response patterns and unresolved measurement concerns. Evaluation of GALS using ordinal factor models and reliability analyses helped align the interpretation of its exploratory score associations with the available measurement evidence.

Several limitations nevertheless constrain interpretation. First, the cross-sectional design cannot establish causality or temporal direction. Communication experiences may influence efficacy beliefs, or both may reflect other personal and workplace conditions. Second, nonprobability recruitment at one training and research hospital limits generalizability. Operating-room nurses were excluded because institutional authorization was not granted, and 60.0% of eligible nurses participated. Participants may have been more interested in communication or professional development; information on nonparticipants was unavailable to assess the direction or magnitude of nonresponse and self-selection bias.

Third, Supplementary Table S1 reports effect sizes with bootstrap 95% confidence intervals for all 36 exploratory subgroup comparisons. Neither a global multiplicity correction nor multiplicity-adjusted intervals were used. Small or unbalanced groups—including postgraduate nurses (n = 6), nurses with less than one year of experience (n = 9), and those familiar with generative altruism (n = 8)—limit interpretation; resampling does not resolve this uncertainty. The initial sample-size calculation concerned a population proportion, and the subsequent regression sensitivity analysis neither established prospective power nor justified subgroup comparisons. These comparisons should therefore be treated as hypothesis-generating.

Fourth, adjustment does not eliminate residual confounding. Education was excluded because of its highly unbalanced distribution, and marital status was omitted after considering age and professional experience. Empathy, workload, staffing, workplace support, and organizational climate were not assessed. Excluding perceived professional competence in a sensitivity analysis did not materially change the association, but this does not exclude construct overlaps or unmeasured confounding.

Fifth, unresolved CCS measurement concerns limit interpretation of the primary association. For each of the 201 participants, original questionnaires confirmed identical CCS responses within four item sets: Items 1–3, 8–10, 12–13, and 19–20. The unusually high Cronbach’s alpha (.994) and these response patterns raise concerns about potential item redundancy and limit interpretation of the CCS total score. Agreement between paper questionnaires and electronic records does not explain the response patterns or establish measurement validity. Electronic NPSE responses were likewise identical within two separate sets (Items 2–4 and 5–9) for every participant. Both scales were completed in the same session, and discriminant validity was not established. Shared response tendencies, social desirability, and overlapping item content may partly account for their association; covariate adjustment and robust standard errors do not address these measurement concerns.

Sixth, sparse or unobserved GALS response categories warrant caution with ordinal estimates; only two categories were observed for Item 5. The restricted score range may have attenuated associations, but range restriction, measurement error, and a weak underlying relationship cannot be distinguished in these data.

## 5. Conclusions

In this single-hospital sample of 201 nurses, NPSE and CCS scores were positively associated after adjustment for selected demographic and professional covariates. However, the unusually high CCS alpha (.994), identical item response patterns, and potential item redundancy leave measurement validity unresolved. Similar estimates across sensitivity analyses do not resolve these concerns. The association should therefore be interpreted at the observed-score level and does not establish causality or differences in independently assessed communication behavior. GALS showed inadequate reliability and factorial validity in this sample. Its exploratory score associations therefore do not support substantive conclusions about generative altruism. Further measurement development and independent validation in adult nurses are needed. The single-hospital, nonprobability sample and 60.0% participation rate limit generalizability. Future multicenter longitudinal studies incorporating independent assessments of communication, followed by controlled intervention research, are needed before educational recommendations can be made. Participants’ interest in training alone does not establish educational need or effectiveness.

## Figures and Tables

**Table 1:** Distribution of participants according to descriptive characteristics (n = 201).

Descriptive Characteristics	Groups	n	%
Age (years) X¯ = 36.90 ± 8.93 (Min = 22, Max = 57)	22–31	67	33.3
	22–31	55	27.4
	≥42	79	39.3
Gender	Female	178	88.6
	Male	23	11.4
Marital Status	Married	137	68.2
	Single	64	31.8
Educational level	Health vocational high school/associate degree Bachelor’s degree Postgraduate degree	21 174 6	10.4 86.6 3.0
Professional experience	<1 year	9	4.5
	1–5 years	41	20.4
	≥6 years	151	75.1
Clinical unit	Internal medicine units *	83	41.3
	Surgical units	57	28.4
	Intensive care/emergency service	61	30.3
Perceived professional competence in nursing	Yes	174	86.6
	No	27	13.4
Previous communication-related training	Yes	65	32.3
	No	136	67.7
Familiarity with the term compassionate communication	Yes	66	32.8
	No	135	67.2
Willingness to receive training in compassionate communication	Yes	162	80.6
	No	39	19.4
Previously heard of generative altruism	Yes	8	4.0
	No	193	96.0
Previous training in generative altruism	Yes	0.0	0.0
	No	201	100.0
Willingness to receive training in generative altruism	Yes	193	96.0
	No	8	4.0
Total		201	100.0

Note: * Internal medicine units included internal medicine, pulmonology, neurology, and other nonsurgical medical wards.

**Table 2:** Mean total scores of the scales among nurses.

Scales (n = 201)	Min	Max	Mean ± SD
Compassionate Communication Scale (CCS)	64	115	91.14 ± 14.63
Generative Altruism Scale (GALS)	3	17	8.35 ± 2.57
Nursing Profession Self-Efficacy Scale (NPSE)	53	78	63.86 ± 6.75

Note: Min and Max refer to observed scores; SD = standard deviation.

**Table 3:** Ordinal confirmatory factor analysis and reliability of the GALS (n = 201).

**Panel A. WLSMV Model-Fit Indices**			
Fit Index		Two-Factor Model	One-Factor Model
Adjusted χ^2^		93.245	119.535
df		26	27
*p*		<0.001	<0.001
CFI, scaled		0.866	0.816
TLI, scaled		0.815	0.754
RMSEA, scaled		0.114	0.131
RMSEA, scaled: 95% CI		0.084–0.144	0.103–0.160
SRMR		0.120	0.133
CFI, robust		0.638	0.508
TLI, robust		0.499	0.344
RMSEA, robust		0.235	0.269
RMSEA, robust: 95% CI		0.178–0.294	0.214–0.326
Latent-factor correlation		0.648	—
**Panel B. Standardized factor loadings**			
Item	Two-factor assignment	Two-factor λ	One-factor λ
GALS1	Factor 1	0.815	0.745
GALS2	Factor 1	0.820	0.770
GALS3	Factor 1	0.345	0.315
GALS4	Factor 2	0.449	0.383
GALS5	Factor 2	0.965	0.879
GALS6	Factor 2	0.759	0.730
GALS7	Factor 2	0.473	0.416
GALS8	Factor 2	0.518	0.488
GALS9	Factor 1	0.163	0.141
**Panel C. Internal consistency**			
Score or item group			Cronbach’s α
GALS total score (Items 1–9)			0.613
Factor 1 item group (Items 1–3 and 9)			0.434
Factor 2 item group (Items 4–8)			0.498

Notes: GALS, Generative Altruism Scale; WLSMV, weighted least squares with mean- and variance-adjusted test statistics; CFI, comparative fit index; TLI, Tucker–Lewis index; RMSEA, root mean square error of approximation; SRMR, standardized root mean square residual; CI, confidence interval; λ, standardized factor loading; α, Cronbach’s alpha. Both models were estimated using WLSMV with all nine items specified as ordered categorical variables (n = 201). Scaled and robust fit indices are reported separately; 95% confidence intervals are reported for RMSEA. Cronbach’s alpha was calculated from the original numerically coded responses. In the two-factor model, Item 5 had a standardized loading of 0.965 (95% CI [0.839, 1.090]); its point estimate was below one, and its residual variance was positive. Only two response categories were observed for this item, warranting caution in interpretation.

**Table 4:** Correlations among the CCS, GALS, and NPSE total scores (n = 201).

Scales	CCS	GALS	NPSE
Compassionate Communication Scale (CCS)	1.000	—	—
Generative Altruism Scale (GALS)	0.028 [−0.111, 0.166] *p* = 0.695	1.000	—
Nursing Profession Self-Efficacy Scale (NPSE)	0.591 [0.493, 0.674] *p* < 0.001	0.123 [−0.016, 0.257] *p* = 0.083	1.000

Notes: Values are Pearson correlation coefficients with 95% confidence intervals in square brackets and two-sided p values. All correlations include 201 participants. Confidence intervals were calculated using Fisher’s z transformation. GALS correlations are exploratory observed-score findings and should not be interpreted as construct-level evidence about generative altruism. CCS measurement concerns also limit interpretation of the NPSE–CCS correlation. CCS, Compassionate Communication Scale; GALS, Generative Altruism Scale; NPSE, Nursing Profession Self-Efficacy Scale.

**Table 5:** Hierarchical multiple linear regression of CCS total scores (n = 201).

**Model 1. Demographic and Professional Covariates**							
**Predictor**	**B**	**SE**	**β**	**95% CI for B**	***p***	**VIF**	**Tolerance**
Constant	99.189	3.058	—	93.158 to 105.220	<0.001	—	—
Age: 32–41 vs. 22–31 years	−2.040	3.067	−0.062	−8.089 to 4.008	0.507	2.521	0.397
Age: ≥42 vs. 22–31 years	−2.810	2.997	−0.094	−8.720 to 3.101	0.350	2.889	0.346
Gender: Male vs. Female	−11.034	2.963	−0.241	−16.879 to −5.189	<0.001	1.200	0.833
Experience: <1 vs. ≥6 years	1.581	5.903	0.022	−10.064 to 13.225	0.789	2.010	0.497
Experience: 1–5 vs. ≥6 years	−9.888	3.451	−0.273	−16.695 to −3.081	0.005	2.608	0.383
Unit: Surgical vs. medical units	4.205	2.107	0.130	0.050 to 8.361	0.047	1.216	0.823
Unit: ICU/emergency vs. medical units	1.665	2.532	0.052	−3.330 to 6.660	0.512	1.828	0.547
Perceived competence: No vs. Yes	−11.026	3.769	−0.258	−18.460 to −3.591	0.004	2.228	0.449
Communication training: No vs. Yes	−5.010	2.018	−0.161	−8.990 to −1.030	0.014	1.201	0.832
R^2^ = 0.335; adjusted R^2^ = 0.304; overall F (9, 191) = 10.698, p < 0.001.							
**Model 2. Covariates + NPSE (primary adjusted model)**							
**Predictor**	**B**	**SE**	**β**	**95% CI for B**	***p***	**VIF**	**Tolerance**
Constant	28.694	8.568	—	11.794 to 45.595	<0.001	—	—
Age: 32–41 vs. 22–31 years	−0.224	2.614	−0.007	−5.380 to 4.933	0.932	2.537	0.394
Age: ≥42 vs. 22–31 years	−2.194	2.547	−0.073	−7.219 to 2.830	0.390	2.891	0.346
Gender: Male vs. Female	−8.627	2.533	−0.188	−13.624 to −3.630	<0.001	1.215	0.823
Experience: <1 vs. ≥6 years	5.590	5.038	0.079	−4.347 to 15.527	0.269	2.027	0.493
Experience: 1–5 vs. ≥6 years	−6.808	2.954	−0.188	−12.635 to −0.981	0.022	2.646	0.378
Unit: Surgical vs. medical units	3.756	1.791	0.116	0.224 to 7.288	0.037	1.217	0.822
Unit: ICU/emergency vs. medical units	2.906	2.156	0.092	−1.348 to 7.159	0.179	1.836	0.545
Perceived competence: No vs. Yes	−11.092	3.203	−0.259	−17.410 to −4.775	<0.001	2.228	0.449
Communication training: No vs. Yes	−1.235	1.769	−0.040	−4.725 to 2.255	0.486	1.280	0.782
NPSE	1.032	0.119	0.476	0.796 to 1.267	<0.001	1.210	0.827
R^2^ = 0.522; adjusted R^2^ = 0.497; overall F(10, 190) = 20.790, *p* < 0.001. Change from Model 1: ΔR^2^ = 0.187; F-change(1, 190) = 74.550, *p* < 0.001.							
**Model 3. Covariates + NPSE + GALS (exploratory extension)**							
**Predictor**	**B**	**SE**	**β**	**95% CI for B**	***p***	**VIF**	**Tolerance**
Constant	27.605	8.562	—	10.716 to 44.494	0.001	—	—
Age: 32–41 vs. 22–31 years	−0.644	2.617	−0.020	−5.807 to 4.519	0.806	2.563	0.390
Age: ≥42 vs. 22–31 years	−2.867	2.572	−0.096	−7.941 to 2.207	0.266	2.972	0.336
Gender: Male vs. Female	−9.120	2.542	−0.199	−14.135 to −4.105	<0.001	1.233	0.811
Experience: <1 vs. ≥6 years	5.204	5.024	0.074	−4.706 to 15.113	0.302	2.032	0.492
Experience: 1–5 vs. ≥6 years	−6.953	2.944	−0.192	−12.760 to −1.147	0.019	2.649	0.378
Unit: Surgical vs. medical units	3.911	1.786	0.121	0.388 to 7.435	0.030	1.220	0.819
Unit: ICU/emergency vs. medical units	1.704	2.278	0.054	−2.789 to 6.198	0.455	2.065	0.484
Perceived competence: No vs. Yes	−11.755	3.217	−0.275	−18.101 to −5.408	<0.001	2.266	0.441
Communication training: No vs. Yes	−0.638	1.802	−0.020	−4.193 to 2.917	0.724	1.338	0.747
NPSE	0.988	0.122	0.456	0.748 to 1.229	<0.001	1.273	0.785
GALS	0.519	0.328	0.091	−0.128 to 1.166	0.115	1.326	0.754
R^2^ = 0.529; adjusted R^2^ = 0.501; overall F(11, 189) = 19.278, *p* < 0.001. Change from Model 2: ΔR^2^ = 0.006; F-change (1, 189) = 2.507, *p* = 0.115.							

Notes: The same 201 participants were included in all models. The dependent variable was the CCS total score. Covariates were entered in Block 1, NPSE in Block 2, and GALS separately in Block 3. The block structure was revised to isolate the incremental contribution of NPSE. Reference categories: age 22–31 years; female gender; ≥6 years of professional experience; medical units; perceived professional competence (yes); and previous communication-related training (yes). B, unstandardized coefficient; SE, standard error; β, standardized coefficient; CI, confidence interval; VIF, variance inflation factor; CCS, Compassionate Communication Scale; NPSE, Nursing Profession Self-Efficacy Scale; GALS, Generative Altruism Scale; ICU, intensive care unit; —, not applicable. Coefficients, confidence intervals, and p values use conventional ordinary least-squares standard errors. GALS was included only as an exploratory observed score because its measurement properties were inadequate. The NPSE–CCS coefficients describe observed-score associations and remain subject to the CCS measurement limitations.

## Data Availability

The data presented in this study are available on request from the corresponding author. The data are not publicly available due to privacy and ethical restrictions.

## Supplementary Materials

The following supporting information can be downloaded at: https://www.mdpi.com/article/10.3390/healthcare14213608/s1. Table S1 presents exploratory subgroup comparisons, including descriptive statistics, test results, and effect sizes with 95% bootstrap confidence intervals. Supplementary File S2 provides the GALS scoring and analysis specifications, item response distributions, item covariance matrix, reliability estimates, results of one- and two-factor ordinal confirmatory factor analyses using mean- and variance-adjusted weighted least squares (WLSMV) estimation, standardized factor loadings, and R code for reproducing the analyses.

## Abbreviations

The following abbreviations are used in this manuscript:

CCS	Compassionate Communication Scale
CFA	Confirmatory Factor Analysis
CFI	Comparative Fit Index
CI	Confidence Interval
GALS	Generative Altruism Scale
HC3	Heteroscedasticity-Consistent Covariance Matrix Estimator, Type 3
ICU	Intensive Care Unit
NPSE	Nursing Profession Self-Efficacy Scale
RMSEA	Root Mean Square Error of Approximation
SRMR	Standardized Root Mean Square Residual
STROBE	Strengthening the Reporting of Observational Studies in Epidemiology
TLI	Tucker–Lewis Index
VIF	Variance Inflation Factor
WLSMV	Weighted Least Squares with Mean- and Variance-Adjusted Test Statistics

## References

[1] Grant J.B., Jones J., Candrian C., Oman K.S., Reed S.M. (2025). The role of nursing communication: A critical interpretive synthesis. Int. J. Nurs. Stud. Adv..

[2] Mielke K., Frerichs W., Cöllen K., Lindig A., Härter M., Scholl I. (2024). Perspective on patient-centered communication: A focus group study investigating the experiences and needs of nursing professionals. BMC Nurs..

[3] Akpinar H., Ozcan Mircali F., Bulut Ugurlu N. (2025). The relationship between therapeutic communication skills and spiritual care competencies of nurses: The example of Türkiye. J. Relig. Health.

[4] Chen Y., Xie Y., Zheng H., Zeng Y. (2022). Altruism in nursing from 2012 to 2022: A scoping review. Front. Psychiatry.

[5] Varghese A., Khakha D.C. (2025). Attributes of compassionate care in clinical nursing practice from the perspectives of patients and nurses—A systematic review. J. Educ. Health Promot..

[6] Efstathiou N., Ives J. (2018). Compassionate care during withdrawal treatment: A secondary analysis of ICU nurses’ experiences. Nurs. Ethics.

[7] Su J.J., Masika G.M., Paguio J.T., Redding S.R. (2020). Defining compassionate nursing care. Nurs. Ethics.

[8] Valizadeh L., Zamanzadeh V., Dewar B., Rahmani A., Ghafourifard M. (2018). Nurse’s perceptions of organisational barriers to delivering compassionate care: A qualitative study. Nurs. Ethics.

[9] İbrahimoğlu Ö., Mersin S., Çağlar M. (2021). The Turkish adaptation of the Compassionate Communication Scale: A validity and reliability study. Perspect. Psychiatr. Care.

[10] Ramos Salazar L. (2013). Communicating with Compassion: The Exploratory Factor Analysis and Primary Validation Process of the Compassionate Communication Scale. Ph.D. Thesis.

[11] Pehlivan T., Güner P. (2020). Compassionate care: Benefits, barriers and recommendations. J. Psychiatr. Nurs..

[12] Pehlivan T., Güner P. (2020). Compassionate care: Can it be defined, provided, and measured?. J. Psychiatr. Nurs..

[13] Tehranineshat B., Rakhshan M., Torabizadeh C., Fararouei M. (2019). Nurses’, patients’, and family caregivers’ perceptions of compassionate nursing care. Nurs. Ethics.

[14] Kacaroğlu Vicdan A., Taştekin A. (2019). A study of adapting the Nursing Profession Self-Efficacy Scale to Turkish. Acıbadem Univ. Sağlık Bilim. Derg..

[15] Caruso R., Pittella F., Zaghini F., Fida R., Sili A. (2016). Development and validation of the Nursing Profession Self-Efficacy Scale. Int. Nurs. Rev..

[16] Durmus K., Akpinar H. (2025). Investigation of the relationship between nurses’ levels of mental health literacy, holistic nursing competencies, and professional self-efficacy. World J. Psychiatry.

[17] Hussien R.M., Khalil T., Nashwan A.J., Al-Najjar H., Khedr M.A. (2025). Compassionate care in nursing: The role of simulation-based compassionate care on nurses’ caring behavior, self-efficacy, and compassion competency. Nurse Educ. Pract..

[18] Takeuchi Y., Tanaka K., Wada T., Seto M., Fujimura K., Kato M., Isihara T. (2026). Characteristics of nursing profession self-efficacy and associated factors: A multiple regression analysis. BMC Nurs..

[19] Kocatepe V., Erkin Ö., Balım B., Şehitoğlu Alpağut G. (2025). The correlation between nurses’ altruism states and compassion fatigue. Celal Bayar Univ. Health Sci. Inst. J..

[20] Büssing A., Kerksieck P., Günther A., Baumann K. (2013). Altruism in adolescents and young adults: Validation of an instrument to measure generative altruism with structural equation modeling. Int. J. Child. Spiritual..

[21] Moura H.M., Filgueiras A., Figueiredo C.V. (2020). Translation and content validation of the Generative Altruism Scale—Brazilian version. Psicol. Conoc. Soc..

[22] Anlı G. (2019). Üretken Özgecilik Ölçeği Türkçe Formu’nun psikometrik özelliklerinin incelenmesi. OPUS Uluslar. Toplum Araşt. Derg..

[23] Julia G.J., Romate J., Allen J.G., Rajkumar E. (2024). Compassionate communication: A scoping review. Front. Commun..

[24] Magon A., Conte G., Dellafiore F., Arrigoni C., Baroni I., Brera A.S., Avenido J., De Maria M., Stievano A., Villa G. (2023). Nursing Profession Self-Efficacy Scale—Version 2: A stepwise validation with three cross-sectional data collections. Healthcare.

[25] Cuschieri S. (2019). The STROBE guidelines. Saudi J. Anaesth..

[26] Akbulut Ö. (2021). Determination of sample size in multivariate and different-scale studies. J. Anim. Sci. Prod..

[27] Virtanen P., Gommers R., Oliphant T.E., Haberland M., Reddy T., Cournapeau D., Burovski E., Peterson P., Weckesser W., Bright J. (2020). SciPy 1.0: Fundamental algorithms for scientific computing in Python. Nat. Methods.

[28] Uysal İ., Kılıç A. (2022). Normal distribution dilemma. Anadolu J. Educ. Sci. Int..

[29] Büyüköztürk Ş., Çokluk Ö., Köklü N. (2024). Statistics for Social Sciences.

[30] Öncü Öner T., Can Ş. (2018). Biostatistical applications in health. IKCU J. Health Sci. Fac..

[31] Arpacı R., Karasu F., Ayar D. (2024). Exploring the link between emotional intelligence and compassionate communication in nurses: A cross-sectional study. West. J. Nurs. Res..

[32] Güdük Ö., Özaydın Ö., Vural A. (2022). Examination of the level of compassion in nurses and nursing students. Gevher Nesibe J. Med. Health Sci..

[33] van den Berg N.M., van der Wal-Huisman H., van Leeuwen B.L., Paans W., Schroevers M.J. (2025). Enablers and barriers of compassionate care from a nurses’ perspective: A scoping review. Scand. J. Caring Sci..

[34] Kıvanç Ö., Yalın H., Eti Aslan F. (2025). The relationship between nurses’ altruism and compassion levels and care quality indicators. Nurs. Forum.

[35] Weil-Lotan D., Danino E., Solnica A. (2026). Shaping professional identity in nursing: A comparison of nurse educators and clinical nurses. Nurse Educ..

[36] Bakhtiari-Dovvombaygi H., Rahmaty Z., Bahramnezhad F. (2026). Investigating the relationship between self-efficacy and caring behaviors in critical care nurses: A cross-sectional study. Creat. Nurs..

[37] Al Wali J., Muthuveloo R., Teoh A.P. (2022). Unravelling the nexus between creative self-efficacy, humble leadership, innovative work behaviour and job performance amongst physicians in public hospitals. Asia Pac. J. Bus. Adm..

[38] Gupta S., Kola N. (2024). Relationship between altruism and self-efficacy among young adults. Int. J. Indian Psychol..

[39] Alavi A., Zargham-Boroujeni A., Yousefy A., Bahrami M. (2017). Altruism, the values dimension of caring self-efficacy concept in Iranian pediatric nurses. J. Educ. Health Promot..

[40] Slettmyr A., Schandl A., Arman M., Hugelius K. (2026). Professional altruism in nursing care: A concept clarification study. Int. J. Nurs. Stud. Adv..

[41] Soyaslan B.D., Öksüz E. (2025). Examination of altruism and compassionate communication levels in nursing students: A cross-sectional study. Scand. J. Caring Sci..

