# Supplementary material for: Association Between Professional Self-Efficacy and Self-Reported Compassionate Communication Among Nurses: A Cross-Sectional Study with Exploratory Analyses of Generative Altruism Scale Scores
Source: Healthcare (Basel). 2026 Oct 25;14(21):3608. doi: 10.3390/healthcare14213608 (PMC13763238; doi:10.3390/healthcare14213608)
Supplement: Supplementary file 1 [file healthcare-14-03608-s001.zip › healthcare-4609187-supplementary.pdf]

Article

Association between Professional Self-Efficacy and Self-Reported Compassionate Communication among Nurses: A Cross-Sectional Study with Exploratory Analyses of Generative Altruism Scale Scores

Supplementary File S2

GALS scoring and psychometric analyses

This supplement documents the scoring, reliability, and ordinal factor analyses of the Generative Altruism Scale (GALS) for the 201 participants included in the revised manuscript. Neither factor model provided adequate measurement support. Only aggregate results are presented; participant-level records are not included.

Data verification and scoring

All nine item responses were available for all 201 participants after verification against the original paper questionnaires. One omitted electronic response to Item 6 was restored as 0 from its source questionnaire. This was a transcription correction, not imputation. Original responses were coded 0–3 and summed without reverse coding, yielding a theoretical total of 0–27. Observed totals ranged from 3 to 17 (mean 8.35; SD 2.57). No alternative scoring scheme was developed.

Model specification

The published two-factor model assigned Items 1–3 and 9 to Factor 1 and Items 4–8 to Factor 2, with correlated factors. The competing one-factor model included all nine items. Models used lavaan 0.7-2 in R 4.5.3, WLSMV estimation, ordered items, delta parameterization, and latent variances fixed to one. No items were removed, categories combined, or residual covariances added. Thresholds were estimated for observed categories. Models were compared descriptively without a chi-square difference test.

Reliability from original numerically coded responses

Item set	Items	Cronbach's alpha
Total score	1–9	.612573
Published group 1	1, 2, 3, 9	.434092
Published group 2	4–8	.498359

Alpha was calculated as $k/(k-1) \times [1 - \mathrm{tr}(S)/(1'S1)]$, where S is the sample covariance matrix and k is the number of items. These are raw-score alpha estimates, not ordinal alpha estimates based on polychoric correlations. Item-group results do not justify separate substantive subscale interpretations.

Observed response category frequencies

Item	0	1	2	3
1	126	70	5	0
2	109	90	2	0
3	33	130	38	0
4	22	65	111	3
5	169	32	0	0
6	167	33	1	0
7	39	44	112	6
8	82	116	3	0
9	2	3	64	132

Several categories were sparse or unobserved. Item 5 had only two observed categories; Item 6 had one response in category 2. These features warrant caution when interpreting ordinal estimates.

Ordinal model fit

Fit measure	One factor	Two factors
Adjusted χ^2	119.535	93.245
Degrees of freedom	27.000	26.000
CFI scaled	0.816	0.866
TLI scaled	0.754	0.815
RMSEA scaled	0.131	0.114
SRMR	0.133	0.120
χ^2 p	< .001	< .001
RMSEA scaled 95% CI	[0.103, 0.160]	[0.084, 0.144]
CFI robust	0.508	0.638
TLI robust	0.344	0.499
RMSEA robust	0.269	0.235
RMSEA robust 95% CI	[0.214, 0.326]	[0.178, 0.294]

CFI, comparative fit index; TLI, Tucker–Lewis index; RMSEA, root mean square error of approximation; SRMR, standardized root mean square residual. Scaled and robust indices are distinguished. All RMSEA intervals in this supplement are 95% intervals; default 90% intervals from the original console summary are not reproduced.

Standardized factor loadings

Item	One-factor λ	Factor	Two-factor λ	95% CI	p
1	0.745	F1	0.815	[0.675, 0.956]	< .001
2	0.770	F1	0.820	[0.690, 0.950]	< .001
3	0.315	F1	0.345	[0.195, 0.495]	< .001
4	0.383	F2	0.449	[0.308, 0.589]	< .001
5	0.879	F2	0.965	[0.839, 1.090]	< .001
6	0.730	F2	0.759	[0.611, 0.908]	< .001
7	0.416	F2	0.473	[0.311, 0.635]	< .001
8	0.488	F2	0.518	[0.359, 0.676]	< .001
9	0.141	F1	0.163	[-0.010, 0.336]	0.065

The standardized factor correlation was .648. The Item 9 loading was .163 (95% CI [−.010, .336], $p = .065$). The Item 5 loading was .965 (95% CI [.839, 1.090]); the point estimate was below one and the residual variance was positive. The Wald confidence interval extending above one does not represent a negative residual variance in the fitted solution.

Numerical checks and interpretation

Both models converged, passed the lavaan post-estimation check, and produced no recorded warnings or negative residual variances. The polychoric correlation matrix was positive definite (minimum eigenvalue approximately .0764). These numerical checks do not establish good fit or construct validity. GALS associations are exploratory observed-score results and cannot establish substantive relationships involving generative altruism.

Sample covariance matrix

The matrix below uses the original numeric item responses and the N − 1 denominator (N = 201). Values are rounded to six decimal places. It supports verification of raw-score reliability; it is not a polychoric correlation matrix and is not sufficient on its own to reproduce WLSMV estimates, which also require ordinal response information.

Item	1	2	3	4	5	6	7	8	9
1	0.290796	0.142935	0.075050	0.025945	0.066318	0.050348	0.020846	0.072214	0.026244
2	0.142935	0.270199	0.033308	0.057861	0.055174	0.028159	0.131244	0.039726	-0.007289
3	0.075050	0.033308	0.354378	0.108184	0.031020	0.005647	-0.015572	0.054826	0.029453
4	0.025945	0.057861	0.108184	0.500498	0.044378	0.012289	0.149129	0.046692	0.059602
5	0.066318	0.055174	0.031020	0.044378	0.134527	0.082139	0.037338	0.042886	-0.009502
6	0.050348	0.028159	0.005647	0.012289	0.082139	0.154527	0.045995	0.048781	-0.003831
7	0.020846	0.131244	-0.015572	0.149129	0.037338	0.045995	0.695274	0.072040	0.100697
8	0.072214	0.039726	0.054826	0.046692	0.042886	0.048781	0.072040	0.269751	0.030647
9	0.026244	-0.007289	0.029453	0.059602	-0.009502	-0.003831	0.100697	0.030647	0.326318

Item means

Item	1	2	3	4	5	6	7	8	9
Mean	0.398010	0.467662	1.024876	1.472637	0.159204	0.174129	1.422886	0.606965	2.621891

Reproduction requirements

The following R code reads an authorized local copy of the verified SPSS dataset containing variables üöö1–üöö9. The participant-level file is not distributed with this supplement. The code checks completeness and coding, computes totals, sample covariance and raw-score alpha, and fits both ordinal models. Exact reproduction should use the reported R and lavaan versions. The haven package is required to import the SPSS file.

The model-fit and loading results above are transcribed from the verified ordinal analysis report dated 8 October 2026. The sample covariance matrix and raw-score reliability were recalculated from the verified 201-participant dataset when preparing this supplement.

Software documentation

lavaan categorical-data tutorial: https://lavaan.ugent.be/tutorial/cat.html

lavaan fitMeasures documentation: https://search.r-project.org/CRAN/refmans/lavaan/html/fitMeasures.html

Reproduction code 1 of 2

```
library(haven)
library(lavaan)

raw <- read_sav(file.choose())
gals <- as.data.frame(raw[paste0("üöö", 1:9)])
gals[] <- lapply(gals, as.numeric)
names(gals) <- paste0("g", 1:9)
stopifnot(nrow(gals) == 201L, !anyNA(gals),
          all(as.matrix(gals) %in% 0:3))

total <- rowSums(gals)
print(c(n = length(total), mean = mean(total),
        sd = sd(total), min = min(total), max = max(total)))
print(lapply(gals, function(x) table(factor(x, levels = 0:3))))
print(cov(gals), digits = 10)
print(colMeans(gals), digits = 10)

raw_alpha <- function(x) {
  S <- cov(x)
  k <- ncol(x)
  k / (k - 1) * (1 - sum(diag(S)) / sum(S))
}
print(c(
  total = raw_alpha(gals),
  group1 = raw_alpha(gals[c("g1", "g2", "g3", "g9")]),
  group2 = raw_alpha(gals[paste0("g", 4:8)])
))

models <- list(
  one_factor = "F =~ g1 + g2 + g3 + g4 + g5 +
                  g6 + g7 + g8 + g9",
  two_factor = "F1 =~ g1 + g2 + g3 + g9
              F2 =~ g4 + g5 + g6 + g7 + g8
              F1 ~~ F2"
)
```

Reproduction code 2 of 2

```
for (nm in names(models)) {
  cat("\nMODEL:", nm, "\n")
  warnings_seen <- character()
  fit <- withCallingHandlers(
    cfa(models[[nm]], data = gals, ordered = names(gals),
        estimator = "WLSMV", std.lv = TRUE,
        parameterization = "delta"),
    warning = function(w) {
      warnings_seen <<- c(warnings_seen, conditionMessage(w))
    }
  )
  # Request 95%, rather than default 90%, RMSEA intervals.
  fm <- fitMeasures(fit,
                    fm.args = list(rmsea.ci.level = 0.95))
  wanted <- c(
    "chisq.scaled", "df.scaled", "pvalue.scaled",
    "cfi.scaled", "tli.scaled", "rmsea.scaled",
    "rmsea.ci.lower.scaled", "rmsea.ci.upper.scaled",
    "cfi.robust", "tli.robust", "rmsea.robust",
    "rmsea.ci.lower.robust", "rmsea.ci.upper.robust", "srmr"
  )
  print(fm[wanted])
  print(standardizedSolution(fit, ci = TRUE, level = 0.95))
  print(lavInspect(fit, "converged"))
  print(lavInspect(fit, "post.check"))
  print(warnings_seen)
  print(lavInspect(fit, "theta"))
  print(lavInspect(fit, "cor.lv"))
  R <- lavInspect(fit, "sampstat")$cov
  print(eigen(R, symmetric = TRUE)$values)
}
sessionInfo()
```

Article

Association between Professional Self-Efficacy and Self-Reported Compassionate Communication among Nurses: A Cross-Sectional Study with Exploratory Analyses of Genera-ive Altruism Scale Scores

Supplementary Table S1. Exploratory subgroup comparisons and effect sizes (n = 201).

Panel A. CCS scores — Part 1 of 3

Characteristic / group	n	Mean ± SD or median [Q1, Q3][1]	Test statistics and nominal p	Effect size [95% CI][2]
**Age (years)**				
a. 22–31	67	85.09 ± 15.50	Welch F(2, 123.05) = 8.380<br>p < .001	ω² = 0.076<br>[0.018, 0.176]
b. 32–41	55	94.20 ± 13.91		
c. ≥42	79	94.15 ± 12.83		
Post hoc: a < b (adjusted p = 0.002); a < c (adjusted p < .001)				
**Gender**				
Female	178	92.98 ± 13.74	t(199.00) = 5.279<br>p < .001	d = 1.170<br>[0.692, 1.654]
Male	23	76.91 ± 13.70		
**Marital status**				
Married	137	94.71 ± 13.37	Welch t(115.36) = 5.253<br>p < .001	d = 0.817<br>[0.506, 1.161]
Single	64	83.52 ± 14.39		
**Educational level**				
a. High school / associate degree	21	92.00 [91.00, 92.00]	H(2) = 8.313<br>p = 0.016	ε² = 0.042<br>[0.015, 0.085]
b. Bachelor's degree	174	92.00 [89.00, 92.00]		
c. Postgraduate degree †	6	109.50 [95.00, 115.00]		
Post hoc: a < c (adjusted p = 0.012); b < c (adjusted p = 0.044)				

[1] Nonparametric comparisons use median [Q1, Q3]. [2] Group-stratified percentile bootstrap, 10,000 resamples. † n < 10; no substantive subgroup inference. Full notes follow Panel C.

Supplementary Table S1. Exploratory subgroup comparisons and effect sizes (n = 201).

Panel A. CCS scores — Part 2 of 3

Characteristic / group	n	Mean ± SD or median [Q1, Q3][1]	Test statistics and nominal p	Effect size [95% CI][2]
**Professional experience (years)**				
a. <1 †	9	69.00 [69.00, 92.00]	$H(2) = 35.244$<br>$p < .001$	$\varepsilon^2 = 0.176$<br>[0.096, 0.285]
b. 1–5	41	69.00 [69.00, 92.00]		
c. ≥6	151	92.00 [92.00, 99.50]		
Post hoc: a < c (adjusted p = 0.044); b < c (adjusted p < .001)				
**Clinical unit**				
a. Internal medicine	83	92.08 ± 14.33	$F(2, 198.00) = 12.229$<br>$p < .001$	$\omega^2 = 0.100$<br>[0.039, 0.195]
b. Surgical	57	96.93 ± 12.59		
c. ICU / emergency	61	84.46 ± 14.38		
Post hoc: a > c (adjusted p = 0.004); b > c (adjusted p < .001)				
**Perceived professional competence**				
Yes	174	93.66 ± 13.58	$t(199.00) = 6.848$<br>$p < .001$	$d = 1.417$<br>[1.061, 1.812]
No	27	74.96 ± 10.27		
**Previous communication-related training**				
Yes	65	96.28 ± 14.05	$t(199.00) = 3.536$<br>$p < .001$	$d = 0.533$<br>[0.243, 0.843]
No	136	88.69 ± 14.31		

[1] Nonparametric comparisons use median [Q1, Q3]. [2] Group-stratified percentile bootstrap, 10,000 resamples. † n < 10; no substantive subgroup inference. Full notes follow Panel C.

Supplementary Table S1. Exploratory subgroup comparisons and effect sizes (n = 201).

Panel A. CCS scores — Part 3 of 3

Characteristic / group	n	Mean ± SD or median [Q1, Q3][1]	Test statistics and nominal p	Effect size [95% CI][2]
**Familiarity with compassionate communication**				
Yes	66	94.48 ± 13.83	$t(199.00) = 2.287$<br>$p = 0.023$	$d = 0.343$<br>[0.058, 0.642]
No	135	89.51 ± 14.79		
**Willingness to receive compassionate-communication training**				
Yes	162	90.46 ± 14.79	$t(199.00) = -1.348$<br>$p = 0.179$	$d = -0.240$<br>[-0.580, 0.096]
No	39	93.97 ± 13.77		
**Previously heard of generative altruism**				
Yes †	8	92.00 [92.00, 92.00]	$U_1 = 908.0$<br>$p = 0.364$	$r_{r\beta} = 0.176$<br>[0.005, 0.390]
No	193	92.00 [89.00, 92.00]		
**Willingness to receive generative-altruism training**				
Yes	193	92.00 [89.00, 92.00]	$U_1 = 874.5$<br>$p = 0.494$	$r_{r\beta} = 0.133$<br>[-0.242, 0.483]
No †	8	92.00 [73.50, 92.00]		

[1] Nonparametric comparisons use median [Q1, Q3]. [2] Group-stratified percentile bootstrap, 10,000 resamples. † n < 10; no substantive subgroup inference. Full notes follow Panel C.

Supplementary Table S1. Exploratory subgroup comparisons and effect sizes (n = 201).

Panel B. NPSE scores — Part 1 of 3

Characteristic / group	n	Mean ± SD or median [Q1, Q3][1]	Test statistics and nominal p	Effect size [95% CI][2]
**Age (years)**				
a. 22–31	67	62.31 ± 5.64	Welch F(2, 125.17) = 3.585<br>p = 0.031	ω^2 = 0.023<br>[0.000, 0.093]
b. 32–41	55	63.84 ± 6.85		
c. ≥42	79	65.18 ± 7.33		
Post hoc: a < c (adjusted p = 0.023)				
**Gender**				
Female	178	64.37 ± 6.86	Welch t(39.73) = 4.427<br>p < .001	d = 0.673<br>[0.377, 0.957]
Male	23	59.91 ± 4.14		
**Marital status**				
Married	137	65.30 ± 7.23	Welch t(189.72) = 5.592<br>p < .001	d = 0.705<br>[0.451, 0.972]
Single	64	60.77 ± 4.20		
**Educational level**				
a. High school / associate degree	21	60.00 [56.00, 64.00]	H(2) = 14.558<br>p < .001	ε^2 = 0.073<br>[0.039, 0.127]
b. Bachelor’s degree	174	61.00 [58.00, 71.00]		
c. Postgraduate degree †	6	77.00 [75.50, 77.00]		
Post hoc: a < c (adjusted p < .001); b < c (adjusted p = 0.002)				

[1] Nonparametric comparisons use median [Q1, Q3]. [2] Group-stratified percentile bootstrap, 10,000 resamples. † n < 10; no substantive subgroup inference. Full notes follow Panel C.

Supplementary Table S1. Exploratory subgroup comparisons and effect sizes (n = 201).

Panel B. NPSE scores — Part 2 of 3

Characteristic / group	n	Mean ± SD or median [Q1, Q3][1]	Test statistics and nominal p	Effect size [95% CI][2]
**Professional experience (years)**				
a. <1 †	9	59.00 [57.00, 59.00]	H(2) = 9.052<br>p = 0.011	ε^2 = 0.045<br>[0.012, 0.107]
b. 1–5	41	61.00 [58.00, 62.00]		
c. ≥6	151	62.00 [58.00, 71.00]		
Post hoc: no pairwise comparison reached adjusted p < .05.				
**Clinical unit**				
a. Internal medicine	83	64.78 ± 7.27	Welch F(2, 125.43) = 10.688<br>p < .001	ω^2 = 0.066<br>[0.019, 0.146]
b. Surgical	57	65.47 ± 6.86		
c. ICU / emergency	61	61.08 ± 4.92		
Post hoc: a > c (adjusted p = 0.001); b > c (adjusted p < .001)				
**Perceived professional competence**				
Yes	174	64.44 ± 7.00	Welch t(90.33) = 5.820<br>p < .001	d = 0.662<br>[0.451, 0.875]
No	27	60.07 ± 2.76		
**Previous communication-related training**				
Yes	65	66.89 ± 7.18	Welch t(108.71) = 4.354<br>p < .001	d = 0.698<br>[0.371, 1.066]
No	136	62.40 ± 6.04		

[1] Nonparametric comparisons use median [Q1, Q3]. [2] Group-stratified percentile bootstrap, 10,000 resamples. † n < 10; no substantive subgroup inference. Full notes follow Panel C.

Supplementary Table S1. Exploratory subgroup comparisons and effect sizes (n = 201).

Panel B. NPSE scores — Part 3 of 3

Characteristic / group	n	Mean ± SD or median [Q1, Q3][1]	Test statistic and nominal p	Effect size [95% CI][2]
**Familiarity with compassionate communication**				
Yes	66	64.55 ± 7.40	Welch t(114.06) = 0.964<br>p = 0.337	d = 0.152<br>[-0.156, 0.474]
No	135	63.52 ± 6.41		
**Willingness to receive compassionate-communication training**				
Yes	162	63.61 ± 6.60	t(199.00) = -1.047<br>p = 0.296	d = -0.187<br>[-0.570, 0.182]
No	39	64.87 ± 7.37		
**Previously heard of generative altruism**				
Yes †	8	72.50 [59.50, 74.00]	U_1 = 971.0<br>p = 0.217	$r_{r\beta}$ = 0.258<br>[-0.236, 0.683]
No	193	61.00 [58.00, 71.00]		
**Willingness to receive generative-altruism training**				
Yes	193	61.00 [58.00, 71.00]	U_1 = 958.0<br>p = 0.248	$r_{r\beta}$ = 0.241<br>[-0.155, 0.619]
No †	8	59.00 [57.75, 65.00]		

[1] Nonparametric comparisons use median [Q1, Q3]. [2] Group-stratified percentile bootstrap, 10,000 resamples. † n < 10; no substantive subgroup inference. Full notes follow Panel C.

Supplementary Table S1. Exploratory subgroup comparisons and effect sizes (n = 201).

Panel C. GALS scores — Part 1 of 3

Characteristic / group	n	Mean ± SD or median [Q1, Q3][1]	Test statistic and nominal p	Effect size [95% CI][2]
**Age (years)**				
a. 22–31	67	8.78 ± 2.21	$F(2, 198.00) = 1.772$<br>$p = 0.173$	$\omega^2 = 0.008$<br>[0.000, 0.067]
b. 32–41	55	7.91 ± 2.46		
c. ≥42	79	8.29 ± 2.87		
**Gender**				
Female	178	8.16 ± 2.51	$t(199.00) = -2.902$<br>$p = 0.004$	$d = -0.643$<br>[-1.121, -0.195]
Male	23	9.78 ± 2.59		
**Marital status**				
Married	137	7.99 ± 2.67	$t(199.00) = -2.992$<br>$p = 0.003$	$d = -0.453$<br>[-0.752, -0.177]
Single	64	9.12 ± 2.13		
**Educational level**				
a. High school / associate degree	21	9.00 [8.00, 10.00]	$H(2) = 1.861$<br>$p = 0.394$	$\varepsilon^2 = 0.009$<br>[0.001, 0.050]
b. Bachelor's degree	174	8.00 [6.00, 10.00]		
c. Postgraduate degree †	6	9.00 [8.25, 10.50]		

[1] Nonparametric comparisons use median [Q1, Q3]. [2] Group-stratified percentile bootstrap, 10,000 resamples. † n < 10; no substantive subgroup inference. Full notes follow Panel C.

Supplementary Table S1. Exploratory subgroup comparisons and effect sizes (n = 201).

Panel C. GALS scores — Part 2 of 3

Characteristic / group	n	Mean ± SD or median [Q1, Q3][1]	Test statistic and nominal p	Effect size [95% CI][2]
**Professional experience (years)**				
a. <1 †	9	9.00 [8.00, 10.00]	H(2) = 10.587<br>p = 0.005	ε² = 0.053<br>[0.014, 0.124]
b. 1–5	41	9.00 [8.00, 10.00]		
c. ≥6	151	8.00 [6.00, 9.00]		
Post hoc: b > c (adjusted p = 0.017)				
**Clinical unit**				
a. Internal medicine	83	7.92 ± 2.85	Welch F(2, 128.61) = 16.520<br>p < .001	ω² = 0.101<br>[0.044, 0.193]
b. Surgical	57	7.61 ± 2.27		
c. ICU / emergency	61	9.62 ± 1.90		
Post hoc: a < c (adjusted p < .001); b < c (adjusted p < .001)				
**Perceived professional competence**				
Yes	174	8.12 ± 2.61	Welch t(50.05) = -4.591<br>p < .001	d = -0.676<br>[-0.989, -0.391]
No	27	9.81 ± 1.62		
**Previous communication-related training**				
Yes	65	8.65 ± 2.78	t(199.00) = 1.139<br>p = 0.256	d = 0.172<br>[-0.136, 0.482]
No	136	8.21 ± 2.46		

[1] Nonparametric comparisons use median [Q1, Q3]. [2] Group-stratified percentile bootstrap, 10,000 resamples. † n < 10; no substantive subgroup inference. Full notes follow Panel C.

Supplementary Table S1. Exploratory subgroup comparisons and effect sizes (n = 201).

Panel C. GALS scores — Part 3 of 3

Characteristic / group	n	Mean ± SD or median [Q1, Q3][1]	Test statistic and nominal p	Effect size [95% CI][2]
**Familiarity with compassionate communication**				
Yes	66	7.94 ± 2.40	t(199.00) = -1.586<br>p = 0.114	d = -0.238<br>[-0.537, 0.039]
No	135	8.55 ± 2.63		
**Willingness to receive compassionate-communication training**				
Yes	162	8.20 ± 2.59	t(199.00) = -1.635<br>p = 0.104	d = -0.292<br>[-0.627, 0.030]
No	39	8.95 ± 2.39		
**Previously heard of generative altruism**				
Yes †	8	7.50 [4.75, 10.25]	U_1 = 661.0<br>p = 0.489	$r_{r\beta}$ = -0.144<br>[-0.624, 0.349]
No	193	8.00 [7.00, 10.00]		
**Willingness to receive generative-altruism training**				
Yes	193	8.00 [6.00, 10.00]	U_1 = 717.0<br>p = 0.733	$r_{r\beta}$ = -0.071<br>[-0.444, 0.327]
No †	8	8.50 [7.75, 10.25]		

[1] Nonparametric comparisons use median [Q1, Q3]. [2] Group-stratified percentile bootstrap, 10,000 resamples. † n < 10; no substantive subgroup inference. Full notes follow Panel C.

Supplementary Table S1 notes

Notes: All analyses included 201 participants. Values are mean ± SD for parametric comparisons and median [Q1, Q3] for nonparametric comparisons. Quartiles were calculated using linear interpolation. The test used for each comparison is identified in the table. Mann–Whitney U_1 refers to the first listed group; two-sided asymptotic p values incorporate tie and continuity corrections. Kruskal–Wallis tests include tie correction. Post hoc comparisons used Tukey HSD after conventional ANOVA, Games–Howell after Welch ANOVA, and Dunn tests with Bonferroni adjustment after Kruskal–Wallis tests. Pairwise p values were adjusted within each comparison family; no global multiplicity correction was applied across the 36 exploratory comparisons. Effect sizes are pooled-standard-deviation Cohen's d, conventional ANOVA omega-squared (ω^2), rank-biserial correlation ($r_{r\beta}$), and Kruskal–Wallis epsilon-squared (ε^2). Positive d and $r_{r\beta}$ values indicate higher scores in the first listed group. Rank-biserial correlation was calculated as $2U_1/(n_1n_2) - 1$, and ε^2 as $H/(N - 1)$. Conventional ω^2 was calculated as max[0, (SSbetween − (k − 1)MSwithin)/(SStotal + MSwithin)], where k is the number of groups. For Welch ANOVA comparisons, ω^2 is a descriptive conventional ANOVA effect size, not a Welch-derived estimate. Effect-size 95% confidence intervals were calculated using 10,000 group-stratified percentile bootstrap resamples, preserving group sizes (random seed: 20261009). These pointwise intervals were not adjusted for multiplicity and do not directly correspond to the reported hypothesis tests. Their accuracy may be limited in very small groups or discrete distributions. Findings from small or highly unbalanced groups should not be interpreted substantively. GALS comparisons concern observed scores only because the scale's measurement properties were inadequate in this sample.

Abbreviations: CCS, Compassionate Communication Scale; NPSE, Nursing Profession Self-Efficacy Scale; GALS, Generative Altruism Scale; ICU, intensive care unit; SD, standard deviation; CI, confidence interval; Q1/Q3, first/third quartile.
